# Physical Activity, Sedentary Behavior, Sleep and Self-Regulation in Spanish Preschoolers during the COVID-19 Lockdown

**DOI:** 10.3390/ijerph18020693

**Published:** 2021-01-15

**Authors:** Alicia M. Alonso-Martínez, Robinson Ramírez-Vélez, Yesenia García-Alonso, Mikel Izquierdo, Antonio García-Hermoso

**Affiliations:** 1 Department of Health Sciences, Public University of Navarra, CIBER of Frailty and Healthy Aging (CIBERFES), Instituto de Salud Carlos III, 31006 Pamplona, Spain; aliciamaria.alonso@unavarra.es (A.M.A.-M.); robin640@hotmail.com (R.R.-V.); mikel.izquierdo@gmail.com (M.I.); 2 Navarrabiomed, Complejo Hospitalario de Navarra (CHN), Universidad Pública de Navarra (UPNA), IdiSNA, 31008 Pamplona, Spain; yesenia.garcia.ihs@gmail.com; 3 Laboratorio de Ciencias de la Actividad Física, el Deporte y la Salud, Facultad de Ciencias Médicas, Universidad de Santiago de Chile, USACH, 71783-5 Santiago, Chile

**Keywords:** coronavirus, quarantine, healthy lifestyle, mental health

## Abstract

Background: A better understanding of the effects of the lockdown on lifestyle behaviors may help to guide the public health response to COVID-19 at a national level and to update the global strategy to respond COVID-19 pandemic. The aim of the study was to examine the effects of the COVID-19 lockdown on device-measured physical activity (PA), sedentary time, sleep and self-regulation; and to determine whether PA and sleep are related to self-regulation problems during the lockdown. Methods: PA, sedentary time and sleep were assessed using accelerometry in the week in which the Spanish national state of alarm was declared (*n* = 21). Parents reported preschooler’s self-regulation difficulties (internalizing and externalizing) before (*n* = 268) and during the lockdown (*n* = 157) by a validated questionnaire. Results: Preschoolers showed a decrease in total PA (mean difference [MD] = −43.3 min per day, 95% CI −68.1 to −18.5), sleep efficiency (MD = −2.09%, 95% CI −4.12 to −0.05), an increase in sedentary time (MD = 50.2 min per day, 95% CI 17.1 to 83.3) internalizing (MD = 0.17, 95% CI 0.06 to 0.28) and externalizing (MD = 0.33, 95% CI 0.23 to 0.44) problems. Preschoolers who met the World Health Organization recommendations for PA had lower internalizing scores than non-active peers (MD = −1.28, 95% CI −2.53 to −0.03). Conclusions: Our findings highlight the importance of meeting PA recommendations to reduce psychosocial difficulties during a lockdown situation.

## 1. Introduction

The coronavirus 2 (SARS-CoV-2, COVID-19) is a severe acute respiratory syndrome that first emerged in late 2019. Countries have faced challenges with how to handle the crisis in different ways. Specifically, in March 2020, the Government of Spain declared a national state of alarm to curb the spread of the severe acute respiratory syndrome coronavirus 2 and established a mandatory home “lockdown” from 14 March to 26 April. Accordingly, the coronavirus disease 2019 (COVID-19) pandemic disrupted life for all, with the closure of non-essential businesses and schools. For schoolchildren, this limited the opportunities for movement (i.e., children no longer had access to school-based physical activities such as physical education, recess, and walking to/from school) and social life, disrupting daily schedules and routines. Many children and adolescents were temporarily deprived of institutional, educational environments, social contact with peers and, possibly, adequate cognitive, affective and physical stimuli for their age.

Recent studies published about healthy lifestyles during confinement have shown adverse collateral effects of the COVID-19 lockdown on physical health [1] in children and adolescents. It has been shown that U.S. children performed less physical activity and engaged in more sedentary behavior during the early COVID-19 period as compared to before the pandemic [2]. Another study in Spanish children and adolescents (3 to 16 years old) also reported a reduction in physical activity levels and increased both screen exposure and sleep time [3]. Specifically, these authors suggested that preschoolers (i.e., 3 to 4 years old) reduced their total physical activity (92 min per day) and increased screen time exposure by 2.2 h per day. In contrast, another study showed that during the pandemic Swedish preschooler’s physical activity, time spent outside on weekdays and weekend days, and screen time significantly increased [4]. However, it should be noted that in Sweden, preschools, playgrounds, and parks remained open, and children’s organized sports and activities continued.

Several studies have reported serious changes in the mental health of children and adolescents during the COVID-19 quarantine [5]. For example, research carried out in China informed an increase in depressive, anxiety and stress symptoms during the COVID-19 lockdown [6,7]. A recent narrative review analyzed the impact of COVID-19 and lockdown on the mental health of children and adolescents and suggested that young children show more clinginess, poor appetite, inattentiveness, and significant separation problems [8]. In another study published in Spain, when the families described the observed changes in the psychological well-being of children aged three years old, they mentioned greater difficulties in self-regulation [9]. Self-regulation refers to the ability to control one’s thoughts, behaviors, emotional reactions, and social interactions, even when impulses and urges run contrary to proximal or distal goals [10] and is recognized as an indicator of positive child development [11].

Regarding sleep patterns, studies have shown inconclusive results in the youth population. Overall, the pandemic seems to significantly disturb normal sleep patterns and nightmares for children [8]. The above-mentioned study among Spanish youth reported different results according to age group, showing an increase of sleep time of 0.6 h per day among adolescents (13 to 16 years old) but a reduction of 0.4 h per day in preschoolers [3]. Pietrobelli et al. also showed an increase of 0.65 h per day of sleep time among obese Italian children [1].

A better understanding of the effects of the lockdown on physical activity, sleep and mental health may help to develop suitable strategies as part of the COVID-19 pandemic responses. In this regard, the compulsory movement restriction meant the prohibition of the movement of children outside their home for many weeks in a row, with no certainty about potentially damaging consequences on their health and well-being. Therefore, the purpose of this study was two-fold: to examine the effects of the COVID-19 lockdown on physical activity, sedentary time, sleep and self-regulation in Spanish preschoolers; and to determine whether device-measured PA and sleep are related to self-regulation difficulties during lockdown.

## 2. Materials and Methods

### 2.1. Design and Population Study

The present study was conducted in a cohort of preschoolers aged 4 to 6 years old from three schools in Pamplona (Spain). Data from baseline assessments (from September to December 2019) and from the second evaluation (from March to April 2020) were included in the study. The Ethics Committee of the Public University of Navarra approved the study protocol (PI-020/19). Before the enrollment in the study, all parents or legal guardians were informed about the purpose of the project and signed informed consent.

### 2.2. Procedures

#### 2.2.1. Physical Activity, Sedentary Behavior and Sleep

Objectively measured physical activity, sedentary time, and sleep were collected using a wrist-worn GENEActiv tri-axial accelerometer attached to the preschooler’s non-dominant wrist over six consecutive days [12]. Raw data were sampled at 87.5 Hz and then reintegrated into 1-s epochs using the GGIR package in R (version 1.10-7) (R Foundation for Statistical Computing, Vienna, Austria), which auto-calibrated the recorded accelerometer signals [13]. Total physical activity was defined as total recorded counts/wear time. The accelerometer counts for light, moderate and vigorous physical activity were coded using previously validated specific cut-points for preschool-aged children [14]. To meet the study inclusion criteria, preschoolers must have worn the monitor for ≥600 min during awake time and an average sleep time ≥ 200 min, each of the six days recorded. We used the World Health Organization (WHO) recommendations of physical activity (≥180 min per day of total physical activity including ≥60 min per day of moderate to vigorous physical activity) and sleep (10 to 13 h per day) [15] to determine its compliance.

In this study, the van Hees et al. [16] sleep algorithm was used to detect sleep and wake between self-reported bedtime and get uptime. This method is based on the variability of the orientation of the accelerometer and classifies each five seconds epoch as either sleep or wake. For the purpose of this study, we used sleep duration (i.e., the difference between sleep onset and offset) and efficiency (i.e., the percent of minutes scored as sleep between onset and offset). This device has been shown to correlate well with polysomnography [16].

For the present purpose, we have used data from 21 children (57.1% boys) who wore the accelerometers in the week in which the state of alarm was declared. For analyses, we used the three days prior to lockdown compared to the three days during the lockdown.

#### 2.2.2. Self-Regulation

We used the child self-regulation and behavior questionnaire (CSBQ), which evaluates subscales of cognitive self-regulation, behavioral self-regulation, and emotional self-regulation, as well as sociability, prosocial behavior, externalizing problems and internalizing problems. Each item requests the respondent to evaluate the general frequency of target behaviors on a scale from 1 (not true) to 5 (certainly true). All subscales contain at least 5 items and have been shown to be reliable in preschoolers [10]. Following recommendations for low-risk and general populations, we employed the two subscale model of the questionnaire [17]: externalizing and internalizing problems. Externalizing behaviors include problems such as attention difficulties, self-regulation deficits, antisocial behaviors, aggression, delinquency, and other “undercontrolled” behaviors. Internalizing behaviors include problems such as social withdrawal, loneliness, sense of inferiority, depression, shyness, anxiety, somatic complaints, and other “overcontrolled” behaviors [18].

For the present study, parents reported preschoolers’ psychosocial difficulties before (*n* = 268, first assessment) and during (*n* = 157, from March to April 2020) the COVID-19 lockdown using an online questionnaire that included the CSBQ [10]. This scale presents a high level of reliability (all Cronbach’s *α* > 0.80).

#### 2.2.3. Confounders

Potential confounders identified in previous literature were included in the analyses: Maternal education level was recorded by asking mothers about the highest level of education, dichotomized as university education and below. Maternal education is a key predictor of other resources within the family that strongly predict children’s well-being [19]. Socioeconomic status was measured according to the level of average income per family unit. Socioeconomic factors seem to be lifestyle determinants during the COVID-19 lockdown in children and adolescents [20]. Finally, the students were weighed and measured in light clothing and barefoot. These measurements were used to calculate the BMI (kg/m^2^), which was computed as the weight (kg) divided by the square of the height (m^2^). BMI seems to be an important factor related to physical activity levels during the COVID-19 lockdown [1].

### 2.3. Statistical Analysis

The data are presented as means (standard deviations, SD) or absolute and relative prevalence (*n* [%]). A test of normality of distribution and equality of variance between groups using the Shapiro–Wilk test and Levene’s test were used. All assumptions were met, and an analysis of covariance was employed to evaluate differences before and during the COVID-19 lockdown in physical activity (total and moderate to vigorous physical activity), sedentary time, sleep (duration and efficiency), and self-regulation parameters (externalizing and internalizing scores). Also, we determined differences in self-regulation parameters according to compliance with the WHO recommendations of physical activity and sleep [15]. These analyses were adjusted for age, sex, monthly family income, maternal education, body mass index, and baseline values. Since physical activity and sedentary behavior are codependent, both physical activity parameters (i.e., total and moderate-to-vigorous physical activity) were additionally adjusted by sedentary time, as well as for sedentary time by total physical activity. Results were analyzed with SPSS (version 26.0) (SPSS Inc., Chicago IL, USA), and a *p* < 0.05 was considered statistically significant.

## 3. Results

Table 1 summarizes the characteristics of the sample of preschoolers. There were differences in baseline characteristics between preschoolers that participated or not in the lockdown evaluation only in the number of boys (*p* = 0.002) and in maternal education (*p* < 0.001).

During the lockdown, preschoolers showed a decrease in total physical activity (mean difference (MD) = −43.3 min per day, 95% confidence interval (CI) −68.1 to −18.5) and sleep efficiency (MD = −2.09%, 95% CI −4.14 to −0.04), and an increase in sedentary time (MD = 50.2 min per day, 95% *CI* 17.1 to 83.3) and internalizing (MD = 0.17, 95% CI 0.06 to 0.28) and externalizing (MD = 0.33, 95% CI 0.23 to 0.44) problems (Table 2).

Preschoolers who met the recommendations for physical activity had lower internalizing scores than non-active peers (MD = −1.28, 95% CI −2.53 to −0.03, *p* = 0.046), but not for externalizing scores (MD = −0.61, 95% CI −1.96 to 0.74, *p* = 0.300). Regarding sleep, there were no differences between preschoolers who met or not sleep recommendations (internalizing, MD = −0.03, 95% CI −1.06 to 0.99, *p* = 0.940; externalizing, MD = 0.06, 95% CI −0.69 to 0.82, *p* = 0.839).

## 4. Discussion

The current study explored the effects of the COVID-19 lockdown on physical activity, sedentary behavior and sleep and its relationship with self-regulation difficulties in Spanish preschoolers. Our findings provide evidence of the negative effects of the COVID-19 lockdown on physical activity level, sedentary behavior, sleep quality and self-regulation in Spanish preschoolers. As far as we know, this is the first study that objectively examines the effect of COVID-19 home confinement on these parameters among preschoolers.

Regarding physical activity and sedentary behavior, our study reflects that preschoolers reduced their total physical activity (MD = −43.3 min per day) and increased sedentary time (MD = 50.2 min per day) during the lockdown. Previously published studies are in line with our findings, which found that children had different patterns of activity than what was seen before COVID-19. For example, changes in physical activity and sedentary behavior were reported by parents and legal guardians of children living in the U.S. using an online survey [2]. Among Spanish youth, the lockdown substantially reduced physical activity levels (MD = −102.5 min per week) and increased daily hours of screen time (MD = 2.9 h per day); suggesting that restrictive mobility measures with the closure of schools and high schools had played an important role in these lifestyle behaviors worsening [3]. With higher time spent at home, it can be expected that screen time could reach higher levels than before the COVID-19 lockdown. Therefore, our findings support objectively the hypothesis that unfavorable changes in activity behaviors occurred in preschoolers during a non-school lockdown period. Contrariwise, another study in 100 Swedish preschoolers reported an increased in physical activity and time spent outside on weekdays and weekend days during the lockdown, but also increased screen time used (MD = 30 min per day) [4]. These results could be due to the that preschools, playgrounds, and parks in Sweden remained open and children’s organized sports and activities continued and; therefore, preschools changed their routines to have children outside as much as possible. This study also revealed that active play indoors does not seem to replace active play outdoors, resulting in a net decline in reported play-based activity [4].

Because children and adolescents were experiencing changes regarding their usual daily habits, it also appears reasonable to find different sleep patterns. However, the results of this issue are inconclusive. For example, a recent narrative meta-analysis suggested that the pandemic seems to significantly disturb normal sleep patterns and nightmares for children [8]. The study mentioned above among Spanish youth reported different results according to age group, showing an increase of sleep time of 0.6 h per day among adolescents (13 to 16 years old) but a reduction of 0.4 h per day in preschoolers (3 to 4 years old) [3]. Pietrobelli et al. also showed an increase of 0.65 h per day of sleep time among obese Italian children [1]. Consistent with the above-mentioned review, the present research found that preschoolers slightly reduced their sleep efficiency (i.e., the percent of minutes scored as sleep between onset and offset), but not sleep duration. The changes in daily routines, including the lack of social activities with other children, have probably contributed to the sleep quality impairments [21]. Because sleep is a critical part of health for youths, children and families should adjust their sleep schedules to be well-rested and to have appropriate levels of energy to start their day [22].

Self-regulation is defined as psychological conduct that comprises a series of important competencies, such as the ability to control inner states or responses towards thoughts, attention, emotions or even performance [11]. The present study showed that during the lockdown, preschoolers had an increase in internalizing and externalizing problems. Behavioral and emotional problems at this age may potentially set a child on a course of maladaptation [18], and more specifically on a pathway to internalizing (i.e., antisocial behaviors) or externalizing problems (i.e., anxious or depressed behaviors). In accordance with the present research, recent studies have suggested that the pandemic situation entailed a substantial impact on mental health [23]. This finding was also reported by Giménez-Dasí et al. [9]. In this study, families reported overall greater difficulties in emotional regulation (i.e., he/she is more irritable, has more mood swings) in their children aged three years old during the six weeks of strict confinement experienced in Madrid, Spain.

Our study also shows that preschoolers who met the recommendations for physical activity had lower internalizing scores than non-active peers. Therefore, we also highlight the importance of meeting physical activity recommendations in the early years, as it seems to influence aspects related to broad areas of mental health [24]. This result may be explained by the fact that increased physical activity was associated with higher mental health among children and adolescents [24]. In this aspect, it also has recently been shown that children who meet the physical activity guidelines have higher life satisfaction, positive affect [25] and self-regulation [26] compared to inactive peers. The current results are supported by another study [27] showing that engaging in physical activity, particularly vigorous physical activity, had a beneficial association with preschoolers internalizing problems one year later. It is plausible that the activity of higher intensities may be associated with neurochemical pathways that underpin psychosocial factors that may lead to fewer emotional problems during childhood [28].

As far as we are aware, this is the first study to examine physical activity and sleep patterns of preschoolers using objective-measures with accelerometers during the COVID-19 lockdown. Despite efforts of objectively examining physical activity and sleep via actigraphy, there are a number of methodological issues that also need to be considered. A first limitation is that it is possible that changes in behavior outcomes occurred in the initial week of the lockdown, as there were serious alterations in daily life and families struggled to adapt to their new reality. However, because the lockdown continued for several weeks, it is possible that preschoolers returned to normalcy, including their routine of sleep and physical activity. Second, the small sample size with accelerometry data and a short time of track are other important limitations. Third, subjects at such age are dependent on parents’ decisions regarding their lifestyle. Finally, the geographic/urban environment could influence physical activity and sleep patterns during the early years [20].

## 5. Conclusions

In conclusion, recognizing these lifestyle and psychological well-being changes are critical because they may have a lasting impact on preschoolers’ physical and mental health and may help guide future interventions, perhaps by physical activity promotion. Therefore, adopting healthy movement behaviors may help to mitigate the negative effects on preschool children of this pandemic and its lockdown.

## Figures and Tables

**Table 1:** Characteristics of the whole sample of preschoolers participating in the study before the lockdown, and differences in baseline characteristics between preschoolers that participated or not in the lockdown evaluation.

	Whole Sample (*n* = 268)	Preschoolers Not Participating in the Lockdown Evaluation (*n* = 123)	Preschoolers Participating in the Lockdown Evaluation (*n* = 145)	*p*
Sociodemographic characteristics				
Age, years	4.28 (0.80)	4.27 (0.84)	4.29 (0.76)	0.890
Boys, *n* (%)	143 (53.4)	70 (56.9)	73 (46.5)	0.002
Public school, *n* (%)	28 (10.4)	18 (14.6)	10 (6.9)	0.067
Monthly family income ^a^, *n* (%)	102 (38.2)	45 (36.6)	58 (40.2)	0.229
Maternal education ^b^, *n* (%)	146 (43.6)	56 (25.1)	90 (62.1)	<0.001
Anthropometric variables				
Body weight, kg	18.90 (3.33)	19.14 (3.65)	18.64 (2.97)	0.255
Height, cm	107.74 (7.17)	107.88 (7.74)	107.61 (6.57)	0.771
Body mass index, kg/m^2^	16.18 (1.50)	16.33 (1.58)	16.03 (1.40)	0.119
Device-measured physical activity				
Total physical activity, minutes per day	361.3 (67.1)	363.9 (69.0)	346.9 (54.6)	0.351
MVPA, minutes per day	89.0 (31.9)	88.5 (32.8)	91.6 (26.7)	0.684
Sedentary time, minutes per day	620.6 (80.3)	622.4 (82.0)	609.6 (69.4)	0.513
Total wear time, hours	152.3 (25.6)	153.3 (26.7)	146.1 (16.6)	0.233
Meeting recommendations ^c^, *n* (%)	113 (79.0)	95 (77.9)	18 (85.7)	0.131
Device-measured sleep				
Sleep duration, hours per day	9.43 (0.69)	9.42 (0.66)	9.51 (0.74)	0.276
Sleep efficiency, %	84 (0.04)	84 (0.04)	84.3 (4.55)	0.691
Meeting recommendations ^d^, *n* (%)	29 (20.3)	23 (18.9)	6 (28.6)	0.051
Self-regulation				
Internalizing problems (0–5)	1.99 (0.64)	2.17 (0.69)	1.82 (0.59)	0.112
Externalizing problems (0–5)	2.55 (0.46)	2.60 (0.45)	2.51 (0.48)	0.667

Notes: ^a^ More or equal than 3000 euros (€); ^b^ mother with university studies; ^c^ ≥180 min/day of total physical activity including ≥60 min/day of moderate-to-vigorous physical activity; ^d^ 10–13 h/day. MVPA, moderate-to-vigorous physical activity.

**Table 2:** Changes in physical activity, sedentary time, sleep and psychosocial parameters before and during the coronavirus disease 2019 (COVID-19) lockdown in those preschoolers participating in the two evaluations.

	Before the Lockdown	During the Lockdown	Mean Differences (95% CI)	*p* *
Device-measured physical activity (*n* = 21)				
Total physical activity, minutes per day	346.9 (54.6)	303.6 (76.5)	−43.3 (−68.1 to −18.5)	0.002
MVPA, minutes per day	91.6 (26.7)	74.6 (26.0)	−17.0 (−21.7 to −12.4)	<0.001
Sedentary time, minutes per day	609.6 (69.4)	659.8 (116.6)	50.2 (17.1 to 83.3)	0.006
Device-measured sleep (*n* = 21)				
Sleep duration, hours per day	9.51 (0.74)	9.54 (1.30)	0.022 (−0.41 to 0.45)	0.914
Sleep efficiency, %	84.3 (4.55)	82.2 (4.92)	−2.09 (−4.14 to −0.04)	0.047
Self-regulation (*n* = 157)				
Internalizing problems (0–5)	1.82 (0.59)	1.99 (0.68)	0.17 (0.06 to 0.28)	0.003
Externalizing problems (0–5)	2.51 (0.48)	2.85 (0.63)	0.33 (0.23 to 0.44)	<0.001

Notes: CI, confidence interval; MVPA, moderate to vigorous physical activity. * Differences in changes were examined by adjusting for age, sex, monthly family income, maternal education, body mass index, and baseline values. Total and moderate-to-vigorous physical activity were additionally adjusted by sedentary time and sedentary time by total physical activity, respectively.
